# *Kif3a* Deficiency Reverses the Skeletal Abnormalities in *Pkd1* Deficient Mice by Restoring the Balance Between Osteogenesis and Adipogenesis

**DOI:** 10.1371/journal.pone.0015240

**Published:** 2010-12-02

**Authors:** Ni Qiu, Li Cao, Valentin David, L. Darryl Quarles, Zhousheng Xiao

**Affiliations:** 1 Institute of Clinical Pharmacology, Central South University, Changsha, Hunan, China; 2 Division of Nephrology, Department of Medicine, the University of Tennessee Health Science Center, Memphis, Tennessee, United States of America; Pennington Biomedical Research Center, United States of America

## Abstract

Pkd1 localizes to primary cilia in osteoblasts and osteocytes. Targeted deletion of *Pkd1* in osteoblasts results in osteopenia and abnormalities in Runx2-mediated osteoblast development. Kif3a, an intraflagellar transport protein required for cilia function, is also expressed in osteoblasts. To assess the relationship between Pkd1 and primary cilia function on bone development, we crossed heterozygous *Pkd1-* and *Kif3a*-deficient mice to create compound *Pkd1 and Kif3a*-deficient mice. *Pkd1* haploinsufficiency (*Pkd1* ^+/Δ^) resulted in osteopenia, characterized by decreased bone mineral density, trabecular bone volume, and cortical thickness. In addition, deficiency of Pkd1 resulted in impaired osteoblastic differentiation and enhanced adipogenesis in both primary osteoblasts and/or bone marrow stromal cell cultures. These changes were associated with decreased *Runx2* expression, increased *PPARγ* expression, and impaired hedgehog signaling as evidenced by decreased *Gli2* expression in bone and osteoblast cultures. In contrast, heterozygous *Kif3a* ^+/Δ^ mice display no abnormalities in skeletal development or osteoblast function, but exhibited decreased adipogenic markers in bone and impaired adipogenesis *in vitro* in association with decreased *PPARγ* expression and upregulation of *Gli2*. Superimposed *Kif3a* deficiency onto *Pkd1* ^+/Δ^ mice paradoxically corrected the effects of *Pkd1* deficiency on bone mass, osteoblastic differentiation, and adipogenesis. In addition, *Runx2*, *PPARγ* and *Gli2* expression in bone and osteoblasts were normalized in compound double *Pkd1* ^+/Δ^ and *Kif3a* ^+/Δ^ heterozygous mice. The administration of sonic hedgehog, overexpression of *Gli2*, and the PC1 C-tail construct all increased *Gli2* and *Runx2*-II expression, but decreased *PPARγ*2 gene expression in C3H10T1/2 cells. Our findings suggest a role for Pkd1 and Kif3a to counterbalance the regulation of osteogenesis and adipogenesis through differential regulation of Runx2 and *PPARγ* by Gli2.

## Introduction

The primary cilium is a microtubule-based membrane protrusion that is assembled and maintained by the bidirectional intraflagellar transport (IFT) machinery [1] that is involved in the differentiation of mesenchymal stem cells into osteoblasts, chondrocytes and adipocytes. In this regard, disruption of primary cilia results in abnormal skeletal patterning, post-natal growth plate development, and skeletogenesis [2], [3], [4], [5], [6]. Conditional inactivation of kinesin family member 3A (Kif3a), a subunit of kinesin-2 motor complex, in mesenchymal stem cells results in severe patterning defects [3]. Conditional inactivation of *Kif3a* in chondrocytes results in post-natal dwarfism due to premature loss of the growth plate [2], [4]. siRNA-mediated knock down of *Kif3a* in 3T3-L1 preadipocytes also leads to impaired adipocyte differentiation [7]. Primary cilia have also been identified in the osteoblast lineage and have been postulated to play a role in osteoblast differentiation [8], [9].

The mechanisms whereby primary cilia regulate mesenchymal differentiation into the osteoblast lineage have not been defined. Primary cilia house and transport several signaling molecules involved in skeletogenesis and postnatal bone homeostasis [10], [11], [12], [13], including Patched (Ptch1)-Smoothened (Smo)-Hedgehog (Hh)/Gli and polycystins complexes [5], [14], [15]. The Ptch1-Smo-Hh/Gli pathway is initiated by Hh ligand binding to Ptch1 in primary cilia, which releases the inhibition of Smo and allows it to activate Gli transcription factors [15], [16], [17], [18], [19], [20], [21]. Activation of hedgehog signaling and Gli2 results in increased Runx2 expression and osteogenesis, but decreased peroxisome proliferator-activated receptor gamma (PPARγ) expression and adipogenesis [20], [22], [23], [24].

Primary cilia and polycystins are co-expressed in cells within the osteoblast lineage [8] where they have been postulated to regulate skeletogenesis [4], [9], [25], [26], [27]. Although polycystin-1 (PC1), encoded by the *Pkd1* gene, and Polycystin-2 (PC2), encoded by the *Pkd2* gene, are mutated in autosomal dominant polycystic kidney disease [28], [29], [30], [31], loss of polycystin function in mice also causes a severe skeletal phenotype. In this regard, homozygous loss of PC1 is associated with abnormal skeletal development through stimulation of the osteoblast-specific transcription factor *Runx2*-II [8], [33]. Skeletal abnormalities are also observed in heterozygous *Pkd1* mutant mice [8]. Moreover, *Osteocalcin*-Cre mediated conditional deletion of *Pkd1* selectively in the osteoblast lineage results in osteopenia due to decreased osteoblast-mediated bone formation. Conditional deletion of *Pkd1* in osteoblasts also results in increased adipogenesis in bone marrow stromal cell and impaired osteoblast differentiation, indicating that Pkd1 may also play a role in controlling a differentiation switch between the osteoblast and adipocyte lineages [34].

Primary cilia and polycystins are functionally interconnected in many tissues. For example, loss of PC1 or primary cilia in the kidney results in same cystic phenotype. Indeed, polycystic disease can be caused in mouse models by homozygous loss-of-function mutations in proteins required for cilia formation or function, such as TG737, *Kif3a*, fibrocystin, and cystin [35], [36], [37], [38]. Whether polycystins and primary cilia have interdependent functions in skeletogenesis is not known.

In the current study, we sought to examine if PC1 and primary cilia have interdependent functions in osteoblast and bone development. We crossed heterozygous *Pkd1*-deficient mice onto heterozygous *Kif3a*-deficient mice to create double heterozygous *Pkd1* and *Kif3a*-deficient mice in an effort to impair both PC1 and primary cilia function. Unexpectedly, *Kif3a* deficiency upregulated Hh signaling and reversed the effect of mutant *Pkd1* to impair osteoblastic differentiation and stimulate adipogenesis *in vivo* and *in vitro.* These effects on bone development occurred through cross-talk between Pkd1 and Hh pathways at the level of *Gli2* expression in bone and osteoblasts. Thus, we have discovered a new interaction between Hh and Pkd1 components of primary cilia.

## Results

### Confirmation of Pkd1 and Kif3a deficiency in vivo and in vitro

**Figure 1:**
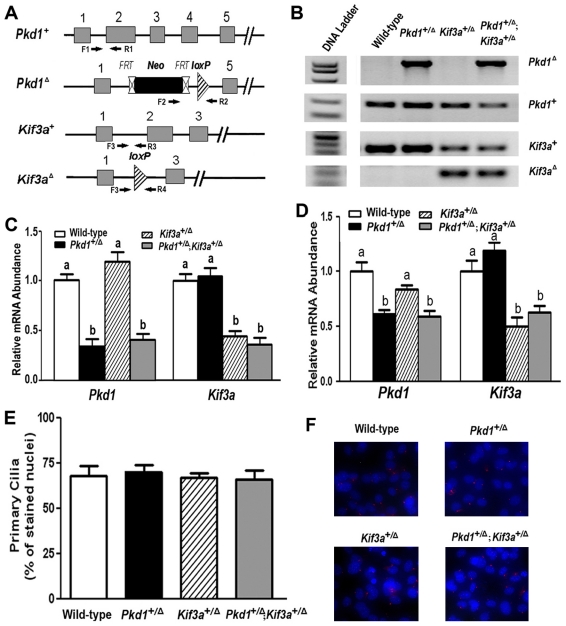
Confirmation of *Pkd1* and *Kif3a* deficiency and *in vivo* and *in vitro*. (A) Schematic illustration of wild-type (*Pkd1* ^+^) and deleted (*Pkd1* ^Δ^) *Pkd1* allele which has been removed the lox P cassette containing Exon 2–4 via Cre-mediated recombination (upper two panels), as well as wild-type (*Kif3a* ^+^) and deleted (*Kif3a* ^Δ^) *Kif3a* allele which has been excised the lox P cassette containing Exon 2 via Cre-mediated recombination (lower two panels). (B) Genotype PCR analysis of tail genomic DNA harvested from different individual mice. Four genotypes were generated in this breeding strategy. (C–D) Real-time RT-PCR analysis of total *Pkd1* and *Kif3a* transcripts from the tibias of 6-week-old mice (C) and the cultured primary osteoblasts (D) by real-time RT-PCR. The level of *Pkd1* or *Kif3a* transcripts exhibited almost ∼50% decreases in long bone samples and primary cultured osteoblasts from single *Pkd1* ^+/Δ^ or *Kif3a* ^+/Δ^ mice, and both *Pkd1* and *Kif3a* transcripts retained the same reductions in compound *Pkd1* ^+/Δ^;*Kif3a* ^+/Δ^ mice compared with wild-type control mice. The value of *Pkd1* or *Kif3a* vs. cyclophilin A from the indicated genotype was a fold difference over wild-type. Data are expressed as the mean±SD from 5 to 6 individual mice. Values sharing the same superscript are not significantly different at *P*<0.05. (E-F) Immunofluorescence of primary cilia in cultured primary osteoblasts. Immunostaining of primary cilia (Red) was performed with acetylated α-tubulin antibody as described in the Materials and Methods. Counterstaining with a nuclear marker (DAPI blue) was used to calculate the percentage presence of primary cilia in cultured primary osteoblasts. There were no obvious number differences in the primary cilia among these four genotype osteoblasts.

Since homozygous *Pkd1* and *Kif3a* null mice are embryonic lethal [32], [39], [40], we examined compound heterozygous *Pkd1* and *Kif3a* deficient mice to establish a potential functional link between Pkd1 and Kif3a. Crossing heterozygous *Pkd1* ^+/Δ^ mice with heterozygous *Kif3a* ^+/Δ^ mice generated four genotypes that were born with the expected Mendelian frequency, including wild-type, heterozygous *Pkd1* ^+/Δ^, heterozygous *Kif3a* ^+/Δ^, and double heterozygous (*Pkd1* ^+/Δ^;*Kif3a* ^+/Δ^) mice (Fig. 1B). The overall survival and body weight of single heterozygous and double heterozygous *Pkd1* ^+/Δ^ and *Kif3a* ^+/Δ^ mice was not different from wild-type littermates. There was no evidence of cyst formation in kidneys of either single or double heterozygous mice (data not shown).

Real-time RT-PCR analysis revealed that the expression of total *Pkd1* or *Kif3a* transcripts from tibias of single heterozygous mice was reduced ∼50% at six weeks of age compared to wild-type control (Fig. 1C), consistent with the deficiency of a single *Pkd1* ^+/Δ^ or *Kif3a* ^+/Δ^ allele. The expression of *Pkd1* or *Kif3a* transcripts in tibias of double heterozygous *Pkd1* ^+/Δ^;*Kif3a* ^+/Δ^ was reduced to the same degree as their respective single heterozygous mice (Fig. 1C). We found that primary osteoblast cultures derived from heterozygous *Kif3a* mice had an approximately 50∼60% reduction in mRNA expression levels of *Kif3a* transcripts (Fig. 1D). Although homozygous disruption of *Kif3a* disables anterograde IFT and leads to failure in the formation and maintenance of cilia [39], heterozygous disruption of Kif3a had no effect on ciliogenesis. Indeed, neither the reduction in *Pkd1* or/and *Kif3a* altered the appearance of primary cilia in cultured osteoblasts (Fig. 1E and 1F).

### Lack of an effect of Kif3a deficiency on skeletogenesis

**Figure 2:**
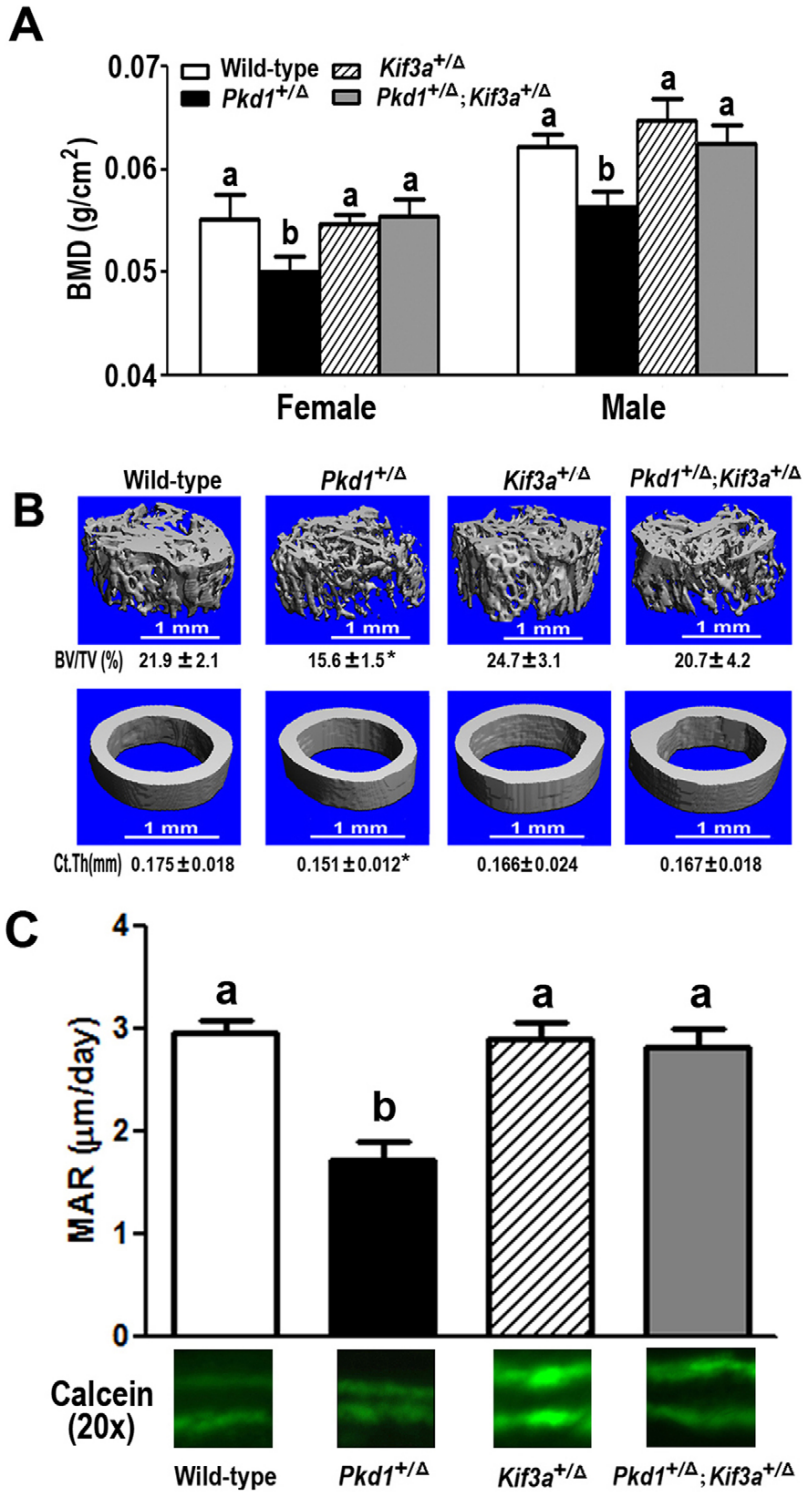
Rescue of the skeletal phenotype in *Pkd1*-deficient mice by transfer onto *Kif3a*-deficient background. (A) Effects of *Pkd1* or/and *Kif3a* on BMD at 6 weeks of age. Compared with age-matched wild-type control, single heterozygous *Pkd1* ^+/Δ^ mice had ∼10% reduction in femoral BMD in both male and female adult mice. In contrast, there were no significant changes in the BMDs in single heterozygous *Kif3a* ^+/Δ^ mice, but the bone mass totally become normal in double heterozygous *Pkd1* ^+/Δ^; *Kif3a* ^+/Δ^ mice, indicating *Kif3a* deficiency predominates over effects of *Pkd1* deficiency on bone mass. The following data only represents male mice. (B) Effects of *Pkd1* or/and *Kif3a* on bone structure of femoral metaphyses and midshaft diaphyses. μCT analysis revealed that single heterozygous *Kif3a* ^+/Δ^ mice exhibited a significant decrease in both BV/TV and CtTh, single heterozygous *Kif3a* ^+/Δ^ mice had no obvious changes in femoral bone structure, and *Kif3a* deficiency corrected the effects of *Pkd1* deficiency on bone structure in compound *Pkd1* ^+/Δ^ and *Kif3a* ^+/Δ^ heterozygous mice. (C) Effects of *Pkd1* or/and *Kif3a* on MAR at 6 weeks of age. Compared with age-matched wild-type, single heterozygous *Pkd1* ^+/Δ^ mice had a significant reduction in periosteal MAR of tibiae. In contrast, there were no significant changes in the tibia MAR in single heterozygous *Kif3a* ^+/Δ^ mice. However, the tibia MAR totally become normal in double heterozygous *Pkd1* ^+/Δ^; *Kif3a* ^+/Δ^ mice. Data are expressed as the mean±SD from 5 to 6 individual mice. Values sharing the same superscript are not significantly different at *P*<0.05. * indicates significant difference from wild type, single heterozygous *Kif3a* ^+/Δ^, and compound heterozygous *Pkd1* ^+/Δ^;*Kif3a* ^+/Δ^ mice at *p<*0.05.

**Figure 3:**
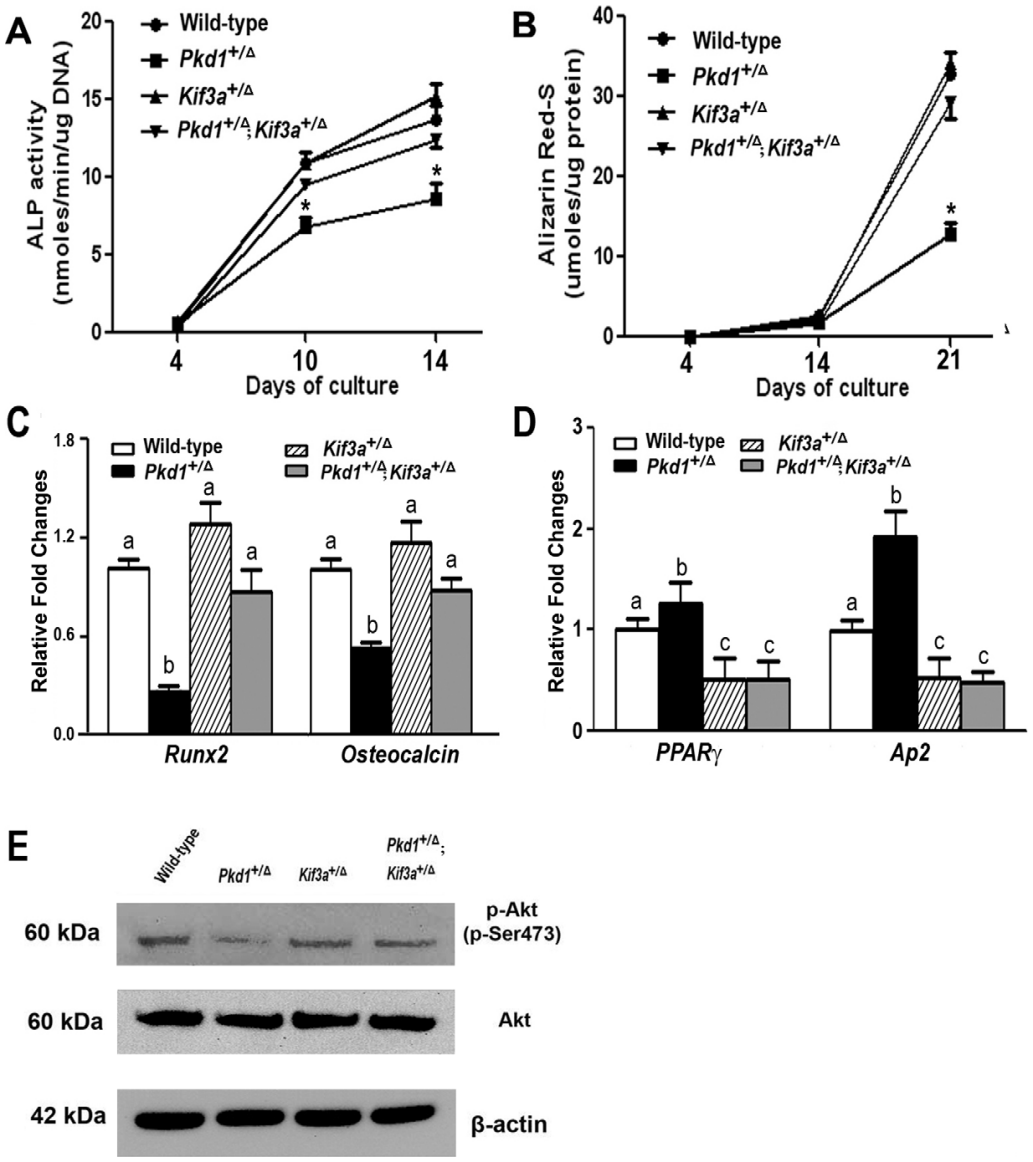
Effects of *Pkd1* or/and *Kif3a* deficiency on osteoblastic differentiation and maturation *ex vivo*. (A) ALP activity. Single *Pkd1* ^+/Δ^ osteoblasts displayed time-dependent increments in ALP activities, but the ALP activity was significantly lower at different time points during 14 days of culture compared with wild-type control. In contrast, single *Kif3a* ^+/Δ^ osteoblasts exhibited normal time-dependent increments in ALP activity and had no difference at the time-matched points with control. However, *Kif3a* deficiency predominated over the effects of *Pkd1* deficiency on ALP activity in compound *Pkd1* ^+/Δ^;*Kif3a* ^+/Δ^ osteoblasts. (B) Quantification of mineralization. Single *Pkd1* ^+/Δ^ osteoblasts had time-dependent increments in calcified nodule formation as measured by Alizarin Red-S staining, but the calcified nodules was significantly lower at 21 days of culture compared with wild-type control. In contrast, single *Kif3a* ^+/Δ^ osteoblasts exhibited normal time-dependent increments in calcified nodule formation and *Kif3a* deficiency predominated over the effects of *Pkd1* deficiency on calcified nodule formation in compound *Pkd1* ^+/Δ^ and *Kif3a* ^+/Δ^ osteoblasts. (C–D) Osteogenic and adipogenic gene expression profiles by real-time RT-PCR. Single *Pkd1* ^+/Δ^ osteoblasts in osteogenic medium showed a significant attenuation in osteogenesis, evidenced by a significant reduction in osteoblastic markers such as *Runx2* and *Osteocalcin* expressions (C), whereas a markedly increase of adipocyte markers such as *PPARγ* and *aP2* (D) was observed when compared with wild-type control. In contrast, there was no obvious difference in osteoblast-lineage markers between single *Kif3a* ^+/Δ^ and wild-type osteoblast cultures, but a significant decrease in adipocyte markers was observed in single *Kif3a* ^+/Δ^ osteoblast cultures compared with wild-type control. *Kif3a* deficiency predominated over the effects of *Pkd1* deficiency on osteogenic differentiation but retained less adipogenesis potentials in compound *Pkd1* ^+/Δ^ and *Kif3a* ^+/Δ^ osteoblast cultures. (E) Western blot analysis. Phosphorylated Akt at Ser473 (panel 1), total Akt (panel 2), and β-actin (panel 3) levels in the cytoplasm from cultured osteoblasts were detected by Western blot. Single heterozygous *Pkd1* ^+/Δ^ cells displayed the decreased phosphorylation of the Akt compared with wild-type control, whereas single heterozygous *Kif3a* ^+/Δ^ had no changes, and compound heterozygous *Pkd1* ^+/Δ^;*Kif3a* ^+/Δ^ cells become normal. Data are expressed as the mean±SD from triple three independent experiments. Values sharing the same superscript are not significantly different at *P*<0.05. * indicates significant difference from wild type, single heterozygous *Kif3a* ^+/Δ^, and compound heterozygous *Pkd1* ^+/Δ^;*Kif3a* ^+/Δ^ mice at *p<*0.05.

We found that heterozygous *Kif3a* ^+/Δ^ mice had no demonstrable bone abnormalities (Fig. 2). Indeed, the *Kif3a* ^+/Δ^ mice had both normal bone mineral density (BMD) and bone structure compared with age-matched wild-type control mice (Fig. 2A and 2B). In addition, bone samples from single heterozygous *Kif3a* ^+/Δ^ mice had no detectable changes in markers of osteoblasts or osteoclasts (Table 1). Primary osteoblasts derived from *Kif3a^+/−^* mice underwent an osteoblast differentiation and mineralization program similar to wild-type derived cells (Fig. 3A–C). *Kif3a* deficiency, however, resulted in significant reductions in adipocyte-related markers, including *adipocyte-specific fatty acid binding protein (aP2), lipoprotein lipase (Lpl)*, and *Adiponectin* in long bone samples (Fig. 3D).

**Table 1:** Gene-expression profiles in 6-week-old mice.

Gene	Accession no.	*Pkd1* ^+/Δ^	*Kif3a* ^+/Δ^	*Pkd1* ^+/Δ^;*Kif3a* ^+/Δ^	*p*-value
**Osteoblast lineage**					
*Runx*2	NM_009820	0.51±0.13*	1.35±0.48	0.93±0.11	0.0049
*Osterix*	NM_130458	0.54±0.13*	1.42±0.13	1.03±0.29	0.0033
*Osteocalcin*	NM_007541	0.52±0.09*	1.45±0.39	1.16±0.21	0.0071
*Opg*	MMU94331	0.67±0.08*	1.23±0.22	1.24±0.19	0.0129
*Rank ligand*	NM_011613	0.57±0.08*	0.97±0.09	1.12±0.21	0.0074
*Dmp1*	MMU242625	0.65±0.11*	0.94±0.18	1.22±0.16	0.0060
**Osteoclast**					
*Trap*	NM_007388	0.29±0.14*	0.68±0.12	0.76±0.22	0.0051
*Mmp*9	NM_013599	0.67±0.11*	0.97±0.14	1.02±0.25	0.0246
**Adipocyte**					
***PPARγ***	NM_009505	1.57±0.24*	1.10±0.27	1.11±0.18	0.0090
***aP2***	NM_024406	0.91±0.14	0.59±0.16#	0.99±0.29	0.0292
***Lpl***	NM_008509	1.31±0.15*	0.76±0.06#	0.92±0.15	0.0003
***Adiponectin***	NM_009605	1.42±0.34*	0.65±0.11#	0.92±0.16	0.0015

Data are mean ±S.D. from 5–6 tibias of 6-week-old individual mice and expressed as the fold changes relative to the housekeeping gene *cyclophilin* A subsequently normalized to wild-type mice.

*indicates significant difference from wild-type, and

# indicates significant difference from single heterozygous *Kif3a* ^+/Δ^, and double heterozygous *Pkd1* ^+/Δ^;*Kif3a* ^+/Δ^ mice at *p<*0.05, respectively.

### Pkd1 deficiency induces osteopenia due to impaired osteoblast differentiation

We observed osteopenia in heterozygous *Pkd1* ^+/Δ^ mice, similar to previously described heterozygous *Pkd1* ^m1Bei^ mutant mice [8]. Indeed, haploinsufficiency of *Pkd1* expression in bone tissues resulted in a ∼10% reduction in femoral BMD in both male and female adult mice (Fig. 2A). Micro-computed tomography (μCT) analysis revealed that the reduction in bone mass in single *Pkd1* ^+/Δ^ mice was caused by a reduction in trabecular bone volume (BV/TV, 29%) and cortical bone thickness (CtTh, 14%) (Fig. 2B). These reductions in bone volume and cortical thickness were associated with a significant decrease in mineral apposition rate in single *Pkd1* ^+/Δ^ mice compared with age-matched control (Fig. 2C).

We also found that low bone formation rates rather than increased bone resorption contributed to ostepenia in *Pkd1* ^+/Δ^ mice. In this regard, bone derived from single heterozygous *Pkd1* ^+/Δ^ mice had significant reductions in the osteoblast-lineage gene transcripts, including *Runx2*, *Osteocalcin*, *Osterix*, *Osteoprotegerin* (*Opg*), *Rank ligand* (*RankL*), and *dentin matrix protein 1 (Dmp1)* mRNA levels compared to wild-type control mice (Table 1). The ratio of Opg/*RankL* that favors reduced osteoclastogenesis, and the bone expression of *tartrate-resistant acid phosphatase (Trap)*, a marker of bone resorption, was reduced in heterozygous *Pkd1* ^+/Δ^ mice (Table 1). In contrast, *PPAR*γ, *Lpl,* and *Adiponectin* but not *aP2,* markers of adipocyte differentiation, were significantly increased in the tibia of heterozygous *Pkd1* ^+/Δ^ mice (Table 1). Serum levels of *Osteocalcin* and TRAP were also reduced in 6-week-old heterozygous *Pkd1* ^+/Δ^ mice compared to wild-type littermates (Table 2).

**Table 2:** Biochemistry analysis of serum in 6-week-old mice.

Genotype	*Wild-type*	*Pkd1* ^+/Δ^	*Kif3a* ^+/Δ^	*Pkd1* ^+/Δ^;*Kif3a* ^+/Δ^
BUN(mg/dl)	20±1.7	18±3.8	23±3.1	19±2.5
Ca (mg/dl)	9.2±0.29	9.1±0.19	9.0±0.21	8.9±0.26
P (mg/dl)	8.1±0.52	7.9±0.67	8.5±0.93	7.8±0.54
Osteocalcin (ηg/ml)	55±2.3	45±3.9*	56±4.1	52±2.9
TRAP (U/L)	2.1±0.18	1.6±0.02*	1.9±0.06	1.9±0.11

Data are mean ±S.D. from 4–6 individual mice.

*indicates significant difference from wild-type, single heterozygous *Kif3a* ^+/Δ^, and double heterozygous *Pkd1* ^+/Δ^;*Kif3a* ^+/Δ^ mice at *p<*0.05, respectively. Osteocalcin is produced by osteoblasts, and TRAP is produced by osteoclasts.

### Rescue of osteopenia, abnormal mineralization and defective adipogenesis associated with Pkd1 deficiency in compound Kif3a and Pkd1 deficient mice

Next, we assessed the effects of superimposed heterozygous deficiency of Kif3a on BMD and bone structure in Pkd1-deficient mice. Surprisingly superimposed *Kif3a* ^+/Δ^ deficiency onto heterozygous *Pkd1* ^+/Δ^ mice corrected the skeletal phenotype observed in single mutant *Pkd1* deficient mice (Fig. 2). Heterozygous deficiency of *Kif3a* in *Pkd1* deficient mice corrects the effects of *Pkd1* deficiency to lower bone mass (Fig. 2A and 2B) and mineral apposition rate (MAR) (Fig. 2C). In addition, compound *Pkd1* ^+/Δ^;*Kif3a* ^+/Δ^ heterozygous mice demonstrated normal expression of the osteoblastic, adipocytic and osteoclastic transcripts in whole bone (Table 1). Both heterozygous *Kif3a* ^+/Δ^ and compound *Pkd1* ^+/Δ^;*Kif3a* ^+/Δ^ heterozygous mice had no significant alterations in serum markers of bone formation and resorption markers at 6 weeks of age compared with wild-type control (Table 2). The apparent rescue of the Pkd1-mutant phenotype was not due to differences in genetic background, since both Pkd1 and Kif3a mutant mice had been crossed onto the C57BL/6J background for multiple generations.

To examine the cellular basis for these alterations in bone, we examined the impact of combined *Pkd1* and *Kif3a* deficiency on cell proliferation, osteoblastic differentiation, and gene expression profiles in cells isolated from calvaria of newborn wild-type, single heterozygous and compound heterozygous *Pkd1* and *Kif3a* deficient mice (Fig. 3). Cells isolated from calvaria are a mixed population capable of differentiation into both osteogenic and adipogenic pathways in culture [41], [42]. We observed no significant changes in 5-bromo-2-deoxyuridine (BrdU) incorporation of calvarial derived cells between the four genotypes (data not shown), indicating neither *Pkd1* nor *Kif3a* deficiency affected cell proliferation. Concordant with defects in both osteoblast and adipocyte developmental markers observed whole bone in vivo (Table 1), we found that cultured calvarial cells derived from single heterozygous *Pkd1* ^+/Δ^ mice displayed lower alkaline phophatase (ALP) activity (Fig. 3A) and diminished calcium deposition in extracellular matrix (Fig. 3B), reduced osteoblastic gene expression markers, such as *Runx2* and *Osteocalcin* (Fig. 3C), and increased adipogenic gene expression, including *PPAR*γ and *aP2* (Fig. 3D) compared to wild-type controls. Calvarial culture derived from heterozygous *Pkd1* ^+/Δ^ also demonstrated decreased phosphorylation of the Akt (Fig. 3E), consistent with our prior results showing coupling of Pkd1 to the PI3K/Akt pathway in osteoblasts [43].

In contrast, single heterozygous *Kif3a* ^+/Δ^ osteoblasts had no abnormalities in ALP activity, mineralization of extracellular matrix, or osteogenic gene expressions profiles (Fig. 3C–3E). Consistent with the decreased adipogenic markers in whole bone of *Kif3a* ^+/Δ^ mice, however, calvarial derived cells from *Kif3a* ^+/Δ^ mice expressed significantly lower levels of *PPAR*γ and *aP2* compared to wild-type controls.

The defective osteoblastic differentiation, mineral deposition, and osteogenic gene expression in calvarial cultures derived *Pkd1-deficient* mice was completely reversed by superimposing *Kif3a* deficiency. Indeed, calvarial cells derived compound heterozygous *Pkd1* ^+/Δ^;*Kif3a* ^+/Δ^ mice had alkaline phosphatase activity, mineralization and expression of Runx2 and osteocalcin transcripts that did not differ from wild-type control cultures (Fig. 3C–3F). Moreover, adipogenic gene expression (i.e., *PPAR*γ and *aP2*) that was increased in *Pkd1* ^+/Δ^ derived calvarial cells was suppressed in compound heterozygous *Pkd1* ^+/Δ^;*Kif3a* ^+/Δ^ cultures to levels not significantly different from *Kif3a* ^+/Δ^ –derived cells (Fig. 3F), consistent with a predominant role of *Kif3a* deficiency to regulate adipogenesis.

**Figure 4:**
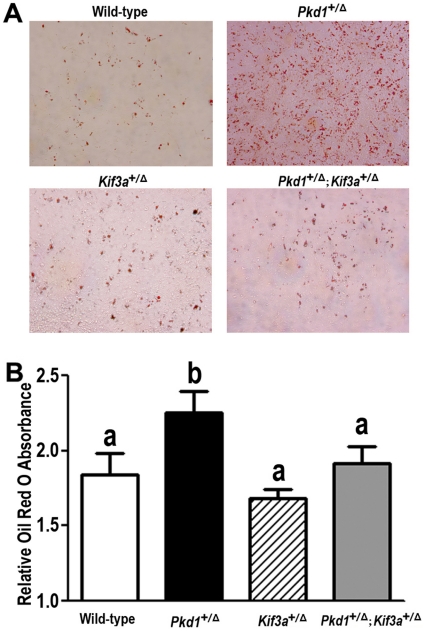
Effects of *Pkd1* or/and *Kif3a* deficiency on adipocytic differentiation in BMSC cultures. (A) Representative cells were stained with Oil Red O in 10-days cultured *Pkd1* ^+/Δ^ or/and *Kif3a* ^+/Δ^ BMSCs as described in Materials and Methods. (B) Relative Oil Red O absorbance from above indicated cultures. Stained lipid was extracted and the absorbance at 510 nm was measured. Clearly, there was a significant increase of adipogenesis potential in single *Pkd1* ^+/Δ^ BMSC cultures, *Kif3a* deficiency entirely reversed the effects of *Pkd1* deficiency on adipogenesis potentials in compound *Pkd1* ^+/Δ^;*Kif3a* ^+/Δ^ BMSC cultures. There was no significant difference in adipocytic differentiation between single *Kif3a* ^+/Δ^ and wild-type BMSC cultures. Data are expressed as the mean±SD from triple three independent experiments. Values sharing the same superscript are not significantly different at *P*<0.05.

To further investigate the effects of *Pkd1* or/and *Kif3a* deficiency on the adipogenesis potential, we investigated the adipogenic potential of bone marrow-derived mesenchymal stem cells (BMSC) derived from wild-type, heterozygous *Pkd1* ^+/Δ^, heterozygous *Kif3a* ^+/Δ^, and double heterozygous (*Pkd1* ^+/Δ^;*Kif3a* ^+/Δ^) mice grown in the presence of rosiglitazone [44]. Under these conditions, BMSC derived from wild-type mice cultures exhibited a low density of Oil Red O staining adipocytes. Single heterozygous *Pkd1* ^+/Δ^ mice exhibited a marked increase in adipogenic cells as evidenced by cells with lipid droplet formation (Fig. 4A–4B). There was no significant difference in adipocyte formation between single *Kif3a* ^+/Δ^ and wild-type BMSC cultures. However, superimposed *Kif3a* deficiency entirely reversed the effects of *Pkd1* deficiency to increase adipocyte formation in BMSC derived from compound *Pkd1* ^+/Δ^;*Kif3a* ^+/Δ^ BMSC cultures (Fig. 4A–4B).

### Role of hedgehog/Gli2 signaling in Pkd1 or/and Kif3a deficiency mice

**Figure 5:**
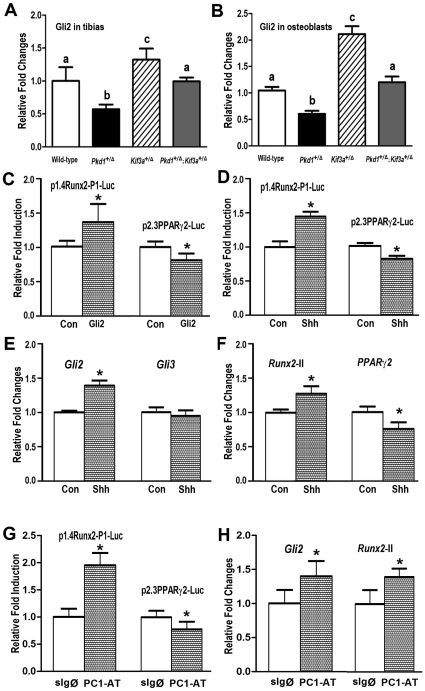
The potential role of hedgehog/Gli2 signaling in *Pkd1* or/and *Kif3a* deficient mice. (A–B) Expression of total *Gli2* transcripts in the tibias (A) and 10 days osteoblast cultures (B) by real-time RT-PCR. A significant reduction of *Gli2* expression was observed in tibias and osteoblasts from single heterozygous *Pkd1* ^+/Δ^ mice when compared with other three groups. In contrast, a markedly increase of *Gli2* expression was found in single heterozygous *Kif3a* ^+/Δ^ mice compared to control and other groups. (C) Effects of *Gli2* on *Runx2*-P1 and *PPARγ*2 promoter activity. Overexpression of Gli2 results in up-regulation of *Runx2*-P1 promoter activity but down-regulation of *PPARγ*2 promoter activity in C3H10T1/2 cells. (D) Effects of Shh on *Runx2*-P1 and *PPARγ*2 promoter activity. Consistent with the effects of Gli2, administration of Shh (1 µg/ml) results in up-regulation of *Runx2*-P1 promoter activity but down-regulation of *PPARγ*2 promoter activity in C3H10T1/2 cells. (E–F) Effects of Shh on *Gli2*, *Gli3*, *Runx2*-II, and *PPARγ*2 expressions in C3H10T1/2 cells. Consistent with promoter activity data, administration of Shh (1 µg/ml) increases *Gli2* and *Runx2*-II transcripts, have no changes in *Gli3 mRNA* message, but decreases *PPARγ*2 gene expression in C3H10T1/2 cell cultures. (G–H) Effects of PC1 C-tail construct on *Gli2*, *Runx2*-II, and *PPARγ*2 expressions in C3H10T1/2 cells. Gain of function PC1 C-tail construct (PC1-AT) stimulated *Runx2*-P1 promoter activity but inhibited *PPARγ*2 promoter activity. Consistent with tibias and primary osteoblasts data, *Gli2* and *Runx2*-II transcripts were significantly increased in C3H10T1/2 cells, which were transiently transfected with PC1-AT for 48 hours. The slgØ construct was served as vector control. Data are expressed as the mean±SD from triple three independent experiments. Values sharing the same superscript are not significantly different at *P*<0.05.

Since heterozygous deficiency of *Kif3a* failed to alter primary cilia formation or Pkd1 expression (Fig. 1E, *vide supra*), we explored the potential role of hedgehog signaling pathway in mediating the effects of Kif3a deficiency to rescue skeletal abnormalities in heterozygous *Pkd1* ^+/Δ^ mice. As a measure of Hedgehog pathway activity in the various mouse models, we assessed expression of total Gli2 transcripts using total RNA from tibias and cultured osteoblasts. We found that Gli2 expression was significantly increased in single heterozygous *Kif3a* ^+/Δ^ mice compared to wild-type controls (Fig. 5A–5B). Although most studies show that mutations in IFT proteins, including Kif3a, result in a down-regulation of Hedgehog signaling [45], [46], [47], inactivation of Kif3a has been reported to paradoxically increase Hedgehog pathway activity in certain tissues, including cranial neural crest cells and the limb [45], [48], [49]. In contrast, we found a significant reduction of Gli2 expression in whole tibias and calvarial-derived osteoblasts from single heterozygous *Pkd1* ^+/Δ^ mice compared to wild-type mice. The effect of Pkd1 deficiency to suppress Gli2 expression was completely reversed in bone and calvarial cultures derived from compound heterozygous *Pkd1* ^+/Δ^;*Kif3a* ^+/Δ^ mice (Fig. 5A and 5B).

C3H10T1/2 cells are multipotent cells capable of developing into osteoblast and adipocyte lineages [44], [50]. To determine if Gli2 mediates the observed effects on osteoblastic and adipocytic differentiation, we overexpressed *Gli2* transcription factor in C3H10T1/2 cells (Fig. 5C). We found that Gli2 significantly stimulates *Runx2*-P1 promoter activity, but suppresses *PPARγ*2 promoter activity in these cells (Fig. 5C). Administration of sonic hedgehog (Shh, 1 µg/ml) resulted in an increase of *Runx2*-P1 promoter activity but decrease of *PPARγ*2 promoter activity in C3H10T1/2 cells (Fig. 5D). C3H10T1/2 cells treated with Shh (1 µg/ml) also displayed increases in *Gli2* and *Runx2*-II gene expression, no changes in Gli3 transcripts, and decrease of *PPARγ*2 message expression (Fig. 5E–5F).To further investigate if PC1 has a direct effect on *Runx2*-P1 and *PPARγ2* promoter activity, we overexpressed gain-of-function PC1 C-tail construct (PC1-AT) in C3H10T1/2 cells (Fig. 5G). We found that PC1-AT significantly stimulates *Runx2*-P1 promoter activity, but suppresses *PPARγ*2 promoter activity in these cells (Fig. 5G). In addition, PC1-AT significantly increases *Gli2* and *Runx2*-II mRNA expression in C3H10T1/2 cells (Fig. 5H), further supporting a role of PC1-Gli2-Runx2 (PPARγ) signaling in osteogenesis and adipogenesis.

## Discussion

We previously reported the presence and co-localization of polycystins and primary cilia in osteoblasts [8] and proposed that polycystins and primary cilia have an interdependent role in regulating osteoblast differentiation and skeletal development [9]. In the current study, we attempted to assess the interaction between Pkd1 and primary cilia by disrupting *Kif3a* in *Pkd1*-deficient mice. We created new mouse models of Pkd1 and Kif3a deficiency by crossing *CMV*-Cre with either *Pkd1* ^flox/+^ mice to achieve a germline deletion of exon 2–4 or *Kif3a* ^flox/+^ mice to attain deletion of exon 2 [51]. Quantitative RT-PCR analysis revealed that the expression of total *Pkd1* transcripts in single *Pkd1* ^+/Δ^ heterozygous mice was reduced by ∼50% compared to wild-type control. We found that this reduction in *Pkd1* expression in heterozygous *Pkd1* ^+/Δ^ mice was sufficient to cause osteopenia due to impairment of osteoblast-mediated bone formation and enhanced adipocyte differentiation. These results are in agreement with our previous reports in heterozygous *Pkd1* ^+/m1Bei^ and conditional *Pkd1* ^Oc-cKO^ null mice [33], [34], indicating that *Pkd1* deficiency favors adipogenesis but inhibits osteogenesis in bone tissues. In contrast, we found that single global *Kif3a* ^+/Δ^ heterozygous mice had no demonstrable bone or kidney phenotype, even though the level of total *Kif3a* transcripts from tibias and cultured osteoblasts was decreased by ∼50%. Hence, haploinsufficiency of Pkd1 had greater effects on bone than partial deficiency of Kif3a.

Our *apriori* assumption was that an interaction between primary cilia and Pkd1 would be revealed by additive effects of Kif3a deficiency to worsen the effects of Pkd1-deficiency to impair bone development in compound *Pkd1* ^+/Δ^;*Kif3a* ^+/Δ^ mice. We found, however, the opposite result. Superimposed Kif3a deficiency in compound *Pkd1* ^+/Δ^;*Kif3a* ^+/Δ^ mice rescued the skeletal abnormalities and defects in osteoblast differentiation observed in *Pkd1* ^+/Δ^ mice. Indeed, reduction of Kif3a in compound *Pkd1* ^+/Δ^;*Kif3a* ^+/Δ^ mice completely reversed the effects of deficiency of Pkd1 on bone mass and osteoblast and adipocyte differentiation, indicating deficiency of Kif3a rescues the bone effects of Pkd1 haploinsufficiency. In addition, neither single *Pkd1* and *Kif3a* nor double *Pkd1* and *Kif3a* mutant mice developed polycystic kidney disease, unlike the reported additive effects of Pkd1 and Pkd2 deficiency to enhance renal cyst formation [52].

This salutary effect of *Kif3a* deficiency was associated with alterations in the osteogenic and adipogenic pathways. Whereas calvarial and bone marrow derived cells from heterozygous *Pkd1* ^+/Δ^ mice had impaired osteoblastic and increased adipocytic differentiation in culture associated with decreased Runx2 and increased PPARγ expression, superimposed *Kif3a* ^+/Δ^ deficiency increased Runx2 and decreased PPARγ expression restoring the normal balance between osteogenic and adipogenic differentiation pathways. Thus, rather than primary cilia and Pkd1 interactions per se, we have shown that two components housed in primary cilia have counter regulatory effects on skeletogenesis.

We have previously shown that loss of Pkd1 inhibits osteoblast development through reductions in intracellular calcium-mediated *Runx2* expression [33] and that primary osteoblasts derived from conditional *Pkd1* ^Oc-Cre^ null mice displayed impaired osteoblastic differentiation and enhanced adipogenesis via suppression of PI3K-Akt-Gsk3β-β-catenin signaling pathway [34]. In the current study, we have found evidence that Hedgehog (Hh)/Gli signaling is a possible mechanism mediating the interactions between *Pkd1* and *Kif3a* on skeletogenesis. Even though partial deficiency of Kif3a did not affect primary cilia number or formation, haploinsufficiency of Kif3a might disrupt IFT necessary for normal hedgehog signaling [18], [45], [51], [53], [54], [55], which in turn could counter the effects of deficiency of Pkd1 signaling [56], [57]. In this regard, Gli2 expression was reduced in both bone and cultured primary osteoblasts from single *Pkd1* ^+/Δ^ heterozygous mice, but was normalized in compound *Pkd1* ^+/Δ^;*Kif3a* ^+/Δ^ heterozygous mice. Since inhibition of microtubule assembly in osteoblasts is reported to stimulate the hedgehog signaling molecule Gli2 expression [58], the increase of Gli2 in single *Kif3a* ^+/Δ^ heterozygous mice may have offset the effects of Pkd1 haploinsufficiency. Indeed, we found that overexpression of gain-of-function PC1 C-tail construct promotes *Gli2* and *Runx2*-II expression, and we also observed that administration of Shh or overexpression of Gli2 up-regulates *Runx2*-P1 promoter activity and expression, consistent with a stimulatory role of Gli2 hedgehog signaling in osteoblast differentiation and bone development [22], [59], [60], [61]. In contrast, either administration of Shh or overexpression of *Gli2* or gain-of-function PC1 C-tail construct down-regulates *PPARγ2* promoter activity and expression, consistent with an inhibitory function of Gli2 hedgehog signaling on adipogenesis and fat formation [22], [23], [62], [63], [64]. In agreement with these findings, knock-down of *Kif3a* by siRNA suppresses adipocyte differentiation in 3T3-L1 preadipocyte cultures [7]. *Kif3a* deficiency reduces expression of PPARγ and CEBPá proteins and has anti-adipogenic role in 3T3-L1 adipogenic cells [7]. Since adipogenesis and osteogenesis are inversely related [65], [66], [67], the reduced adipogenesis in *Kif3a* haploinsufficient mice may have promoted osteoblasts differentiation to offset the effects of deficiency of Pkd1. In support of this hypothesis, we found that Pkd1 and Kif3a haploinsufficiency had opposite effects on adipogenic and osteogenic transcription factors and related gene expressions. *Kif3a* ^+/Δ^ heterozygous mice exhibited significant reductions in adipocyte-related markers such as *PPAR*γ and *aP2*, whereas Pkd1 deficient mice had reductions in osteoblastic markers such as *Runx2* and *Osteocalcin* expression, indicating a counterbalance mechanism involved in *Pkd1* and *Kif3a* haploinsufficient mice. These findings suggest that *Pkd1* or/and *Kif3a* deficiency might affect cell fate decision between osteoblastogenesis and adipogenesis through regulation of hedgehog signaling pathway.

Kif3a mediates intraflagellar transport (IFT), which is necessary for normal formation of the appendicular skeleton through hedgehog signaling pathways [2], [4], [19]. Intraflagellar transport has been shown to be essential for both endochondral bone formation as well as perichondral bone formation, which is a form of intramembranous bone formation. This suggests that IFT may be important in both chondrocytic and osteoblastic lineages, a finding supported by abnormalities of intramembranous bone formation observed in mice after the conditional deletion of *Kif3a* or *Tg737* in mesenchymal skeletal progenitor cells [3], [4], [5], [19]. While most studies suggest that inactivation of *Kif3a* reduces Hh signal transduction [45], [46], [47], [53], Kif3a may also activate Hh signaling pathways in some settings [68], as we observed in heterozygous *Kif3a* ^+/Δ^ mice. For example, conditional deletion of Kif3a in chondrocytes stimulates Indian hedgehog (Ihh) expression in the perichondrium [4]. Also, conditional deletion of *Kif3a* in neural crest cells, which give rise to the facial skeleton, leads to gain of Hedgehog function that results in hypertelorism and iprosopus [48]. Other observations indicate that *Kif3a* and other IFT mutations can have paradoxical effects on hedgehog signaling [45], [49]. This inconsistency has been attributed to the differential effects of Gli proteins (i.e., Gli1, Gli2, or Gli3) that may predominate in different tissues [18], [45], [53], [69].

Another possible explanation for the offsetting effects of Kif3a deficiency to alter the bone phenotype in Pkd1 haploinsufficient mice may be due to Kif3a altering the amount of Pkd1 in primary cilia. This possibility is supported by the fact that Kif3a binds to Pkd2 and regulates the amount of Pkd2 localized in primary cilia [70], [71] and the related observation that the ratio of Pkd1 and Pkd2 determines the net function of the polycystins [72]. Thus, deficiency of Kif3a may lead to a disproportionate reduction in Pkd2 compared to Pkd1 in combined *Kif3a*/*Pkd1* mutant mice, leading to “normalization” of the polycystin signaling by restoring the ratio of Pkd1 and Pkd2 in primary cilia. Consistent with this possibility, transferring *Kif3a*-deficiency onto *Pkd1-*deficiency restores PI3K-Akt signaling in bone and osteoblasts (Fig. 3E).

In summary, Kif3a haploinsufficiency counteracts the negative effects of Pkd1 deficiency on osteoblast function, indicating an interdependent relationship between Pkd1 and Kif3a in postnatal bone formation. While the precise mechanism remains to be defined, *Kif3a* haploinsufficiency up-regulates hedgehog/Gli2 signaling pathways, leading to both increased *Runx2* and osteoblastogenesis and significant inhibition of *PPARγ* and adiopogenesis. In contrast, *Pkd1* haploinsufficiency inhibits hedgehog/Gli2 signaling, *Runx2* expression and osteogenesis but increases *PPARγ* and adiopogenesis. Thus, cross-talk between Pkd1 and Kif3a may play a counterbalancing role on bone formation through the differential regulation of ostoegenesis and adipogenesis in bone. These findings suggest that activation of hedgehog/Gli2 signaling may provide a mechanism to counteract the effects of lost Pkd1 signaling in bone and possibly other tissues, such as the kidney, where defective hedgehog signaling is associated with cystic kidney disease in Oral-facial-digital syndrome type 1 [73]. With regards to our original question of the interdependence of polycystin and primary cilia function in osteoblast development, additional experimental approaches to more completely ablate Kif3a and/or primary cilia in the osteoblasts lineage will be required.

## Materials and Methods

### Mice

We obtained the floxed *Pkd1* mouse (*Pkd1* ^flox/flox^) which harbors two *loxP* sites flanking exon 2–4 of the *Pkd1* gene from Dr. Gregory Germino at Johns Hopkins University [74], the floxed *Kif3a* mouse (*Kif3a* ^flox/flox^) which contains two *loxP* sites flanking exon 2 of the *Kif3a* gene from Lawrence S.B. Goldstein at University of California, San Diego [39], and *CMV*-Cre transgenic mouse from the Jackson Laboratory. We crossed the floxed *Pkd1* ^flox/flox^ or *Kif3a* ^flox/flox^ mouse with *CMV*-Cre mouse to generate global *Pkd1* (*CMV*-Cre;*Pkd1* ^+/Δ^) or *Kif3a* heterozygous (*CMV*-Cre;*Kif3a* ^+/Δ^) mice. The resulting *CMV*-Cre;*Pkd1* ^+/Δ^ or *CMV*-Cre;*Kif3a* ^+/Δ^ mice were then crossed to wild-type mice to segregate the *CMV*-Cre transgene from the desired conditional null allele (*Pkd1* ^Δ^ or *Kif3a* ^Δ^ as shown in Fig. 1A, equivalent to the *Pkd1* or *Kif3a* null allele). These mice were bred and maintained on a C57BL/6J background at least in six generations. Then heterozygous *Pkd1* ^+/Δ^ mice were mated with heterozygous *Kif3a* ^+/Δ^ mice to generate wild-type, heterozygous *Pkd1* ^+/Δ^, heterozygous *Kif3a* ^+/Δ^, and double heterozygous (*Pkd1* ^+/Δ^;*Kif3a* ^+/Δ^) mice. These mice were used for phenotypic analysis. All animal research was conducted according to guidelines provided by the National Institute of Health and the Institute of Laboratory Animal Resources, National Research Council. The University of Tennessee Health Science Center's Animal Care and Use Committee approved all animal studies (Protocol number: 1885R2).

### Genotyping PCR

Genomic DNA was prepared from tail specimens using standard procedures [33]. Genotyping PCR was performed using the following primers as previously described [39], [51], [74]: *Pkd1* wild-type allele, F1, 5′-AAT AGG GGT GGG GCT TGT GGG TCG-3′, R1, 5′-TAC TCA CAC CTC CAC CAG TGC-3′; *Pkd1* conditional null allele, F2, 5′-CGA CCA CCA AGC GAA ACA TC-3′; R2, 5′-TCG TGT TCC CTT ACC AAC CCT C-3′; *Kif3a* wild-type allele, F3, 5′-AGG GCA GAC GGA AGG GTG G-3′, R3, 5′-TCT GTG AGT TTG TGA CCA GCC-3′; *Kif3a* conditional null allele F3, 5′-AGG GCA GAC GGA AGG GTG G-3′, R4, 5′-TGG CAG GTC AAT GGA CGC AG-3′. *Pkd1* wild-type and null alleles were identified in 2% agarose gel as 250-bp and 850-bp bands (Fig. 1B, upper two panels), while *Kif3a* wild-type and null alleles were detected as 360-bp and 200-bp bands (Fig. 1B, lower two panels), respectively.

### Bone Densitometry, histomorphometric and Micro-CT analysis

BMD of femur from 6-week-old mice were scanned using a PIXImus bone densitometer (Lunar Corporation, Madison, WI) with dual-energy X-ray absorptiometry technology. BMD within a defined area of the whole femur was analyzed using proprietary Lunar PIXImus software. Calcein (Sigma, St. Louise, MO) double labeling of bone and histomorphometric analyses of periosteal MAR in tibias were performed using the osteomeasure analysis system (Osteometrics) [75], [76]. Femurs were isolated from 6-week-old mice and fixed in 70% ethanol for μCT analysis. The long axis of the femur was oriented orthogonally to the rotation axis of the scanner. Entire femur scans were performed at an isotropic voxel size of 12 µM using a μCT 40 scanner (Scanco Medical AG, Brüttisellen, Switzerland). A 3D image analysis was done to determine BV/TV in the distal metaphyses and Ct.Th in the midshaft of diaphyses area as previously described [8], [33], [75], [77].

### Quantitative Real-time RT-PCR

A 2.0 µg of total RNA were isolated from tibia of 6-week-old mice or 10 days cultured primary osteoblasts in differentiation media. The cDNAs were generated using a Reverse Transcriptase Kit (Perkin-Elmer, Foster City, CA). PCR reactions contained 100 ηg template (cRNA or cDNA), 200 ηmol each forward and reverse primers, 1X iQ^TM^ SYBR^®^ Green Supermix (Bio-Rad, Hercules, CA) in 50 µl. The threshold cycle (Ct) of tested-gene product from the indicated genotype was normalized to the Ct for cyclophilin A as previously described [8], [33], [77]. Expression of total *Pkd1* transcripts was performed using the following *Pkd1*-specific primers: forward primer of *Pkd1* transcripts in exon 2-4, 5′-TAG GGC TCC TGG TGA ACC TT -3′, and reverse primer of *Pkd1* transcripts in exon 2-4, 5′–CCA GAC CAC AGT TGC ACT CA-3′. Expression of total *Kif3a* transcripts was performed using the following *Kif3a*-specific primers: forward primer of *Kif3a* transcripts in exon 2, 5′-GCT ATA GAC AGG CCG TCA GC-3′, and reverse primer of *Kif3a* transcripts in exon 2, 5′–GTC TTT GGA GGT TCG TTG GA-3′. The value of *Pkd1* or *Kif3a* vs. cyclophilin A from the indicated genotype was normalized to the mean ratio of 5 wild-type mice, which has been set to 1.

### Primary Osteoblast Culture for Proliferation and Differentiation, and western blot analysis

Primary osteoblasts were isolated from the newborn mouse calvarias by sequential collagenase digestion at 37°C as previously described [8], [77]. The cells were cultured in alpha-minimal essential medium (α-MEM) containing 10% fetal bovine serum (FBS) and 1% penicillin and streptomycin (P/S). Cell proliferation was detected by BrdU incorporation assays as the manufacturer describes (QIA58, Calbiochem, Gibbstown, NJ). To induce differentiation, primary osteoblasts were plated at a density of 2.5×10^4^ cells/cm^2^ in a 6-well plate, and grown for period of up to 21 days in α-MEM containing 10% FBS supplemented with 5 mM β-glyceropho-phate and 25 µg/ml of ascorbic acid. Alkaline phosphatase activity and Alizarin red-S histochemical staining for mineralization were performed as previously described [8], [77]. Total DNA content was measured with a PicoGreen® dsDNA quantitation reagent and kit (Molecular Probes, Eugene, OR). Protein concentrations of the supernatant were determined with a Bio-Rad protein assay kit (Bio-Rad, Hercules, CA).

To examine the amounts of cytoplasmic phosphorylated Akt, we isolated cytoplasmic protein using NE-PER Nuclear and Cytoplasmic Extraction Kit (Pierce Biotechnology, Rockford, IL) according to the manufactory’s instruction. Protein concentrations of the supernatant were determined with a Bio-Rad protein assay kit (Bio-Rad, Hercules, CA). Equal quantities of protein were subjected to NuPAGE^TM^ 4–12% Bis-Tris Gel (Invitrogen, Carlsbad, CA) and were analyzed with standard Western blot protocols (HRP-conjugated secondary antibodies from Santa Cruz Biotechnology and ECL from Amersham Biosciences, Buckinghamshire, UK). Antibodies against phospho-Akt (ser-473) and Akt were from Cell Signaling Technology (Beverly, MA). Anti-β-actin (sc-47778) antibodies were from Santa Cruz Biotechnology.

### Transient transfection

Pluripotent C3H10T1/2 mesenchymal cells (American Type Culture Collection, Manassas, VA) were maintained in Eagle’s Minimum Essential Medium (EMEM) containing 10% FBS and 1% P/S. A 2,300 bp promoter region of *PPARγ2* gene was amplified by polymerase chain reaction (PCR) using a set of primers 5′- TCC CCG GGG GTA TGT GG A GCC CA A CCC A -3′ and 5′- ACG TCG ACA GAT TTG CTG TAA TTC ACA CTG G -3′ containing SmaI and SalI sites and the resultant product was subcloned into pluc4 luciferase reporter construct and confirmed by subsequent analysis [78], [79]. To examine if hedgehog signaling regulates *Runx2*-P1 and *PPARγ2* promoter activity, a number of 1×10^6^ of C3H10T1/2 cells were transfected with either *Runx2*-P1 promoter luciferase reporter (p1.4*Runx2*-P1-Luc) or *PPARγ2* promoter luciferase reporter (p2.3*PPARγ2*-Luc) conducted by electroporation using Cell Line Nucleofector Kit R according to the manufacturer's protocol (Amaxa Inc, Gaithersburg, MD). A total of 6.6 µg of plasmid DNA was used for each electroporation, with 3.0 µg of pcDNA3.1 empty expression vector, 3.0 µg of either p1.4*Runx2*-P1-Luc or p2.3*PPARγ2*-Luc reporter, and 0.6 µg of Renilla luciferase-null (RL-null) as internal control plasmid. The cells were cultured in EMEM supplemented with 1%FBS and promoter activity will be assessed by measuring luciferase activity 72 hours after transfection in the presence or absence of 1 µg/ml of recombinant mouse sonic hedgehog N-terminus (Shh-N) treatment. The total RNA was also isolated for real-time RT-PCR analysis. To examine if Gli2 or PC1 has a direct effect on *Runx2*-P1 and *PPARγ2* promoter activity, a *Gli2* or PC1 C-tail (PC1-AT) expression construct along with either p1.4*Runx2*-P1-Luc or p2.3*PPARγ2*-Luc were co-transfected into C3H10T1/2 cells. The cells were cultured for 48 hours in EMEM supplemented with 1%FBS and the relative luciferase activities of cell lysates were measured by a luciferase assay kit (Promega, Madison, WI) and normalized with Renilla luciferase activity and empty expression vector (pcDNA3.1 or sIgØ) as previously described [8], [33]. We also isolated the total RNAs for real-time RT-PCR analysis.

### Immunofluorescence

Primary osteoblasts were grown on collagen-coated 4-well chamber at 1×10^5^ cells per well and kept at confluence for at least 3 days. At the end of the culture, the cells were washed three times with PBS, then fixed with cold 4% paraformaldehyde/0.2% Triton for 10 minutes at room temperature and washed with PBS 3 times. The cells were incubated for 30 minutes in 1% BSA before incubation with the primary acetylated alpha-tubulin antibody (1:4000, Sigma Aldrich, T6793) for 1 hour at room temperature. After washing three times in PBS they were treated with secondary Texas Red-labeled anti-mouse IgG (Jackson ImmunoResearch, 715-076-150) in 1% BSA for 1 hour at room temperature and washed three times in PBS before mounting with ProLong® Gold antifade reagent (Invitrogen, P36935). Nuclei were counter-stained with DAPI blue. Photographs were taken under a microscope with magnifications of 40× for counting the number of primary cilia in cultured primary osteoblasts as previously described [8].

### Adipocyte Differentiation of Bone Marrow-derived Mesenchymal Stem Cells

Primary BMSCs were harvested from the long bone of 8-week-old mice as previously described [75]. Briefly, the epiphysis of long bone was cut off, the bone marrow was flushed out with α-MEM containing 10% FBS and 1% P/S and went through a 70-mm filter mesh. The cells were plated in 100 mm culture dishes at a density of 2.5×10^7^ cells per cm^2^ and cultured in a humidified incubator with 5% CO_2_ and 95% air at 37°C. On day 3, a half of cultured medium was removed and replaced with fresh α-MEM growth medium. On day 5, the adherent cells (representing BMSCs) were detached with 0.25% trypsin/1 mM EDTA and seeded onto 6-well plates at 3×10^4^ cells per well for up to 8 days. To induce adipocyte differentiation, the BMSCs were treated with adipogenic differentiation medium supplemented with 10% FBS, 5 mM beta-glycerophophate and 50 µg/ml of ascorbic acid for 4 days and then added 1 µmol/L rosiglitazone for another 8 days. At the endpoint, the cells were processed for Oil Red O lipid staining as previously described [44]. Breifly, the cells were rinsed with 1xPBS and fixed with cold 4% paraformaldehyde solution for 1 hour at 4°C. The cells were rinsed with distilled water followed by 60% isopropanol for 5 minutes at room temperature. Then the cells were stained in 0.5% Oil Red O-isopropanol working solution for 15 minutes at room temperature, differentiated in 60% isopropanol, and rinsed in tap water. For quantitative analysis, Oil Red O was extracted with 1 ml 99% isopropanol for 2 min, and optical density of each sample was determined at 510 nm.

### Serum Biochemistry

Serum osteocalcin levels were measured using a mouse osteocalcin EIA kit (Biomedical Technologies Inc. Stoughton, MA, USA). Serum urea nitrogen (BUN) was determined using a BUN diagnostic kit from Pointe Scientific, Inc. Serum calcium (Ca) was measured by the colorimetric cresolphthalein binding method, and phosphorus (P) was measured by the phosphomolybdate–ascorbic acid method (Stanbio Laboratory, TX, USA). Serum TRAP was assayed with the ELISA-based SBA Sciences mouseTRAP^TM^ assay (Immunodiagnostic Systems, Fountain Hills, AZ).

### Statistical Analysis

We evaluated differences between two groups by unpaired t-test and multiple groups by one-way analysis of variance. All values are expressed as means ± S.D. All computations were performed using the GraphPad Prism5 (GraphPad Software Inc. La Jolla, CA).
